# Structure-aware deep model for MHC-II peptide binding affinity prediction

**DOI:** 10.1186/s12864-023-09900-6

**Published:** 2024-01-30

**Authors:** Ying Yu, Lipeng Zu, Jiaye Jiang, Yafang Wu, Yinglin Wang, Midie Xu, Qing Liu

**Affiliations:** 1 School of Health Science and Engineering, University of Shanghai for Science and Technology, Shanghai, 200093 China; 2 Department of Computer Science, Florida State University, Tallahassee, 32306 USA; 3 Department of Pathology, Fudan University, Shanghai Cancer Center, Shanghai, 200032 China; 4 Department of Medical Oncology, Shanghai Medical College, Fudan University, Shanghai, 200032 China; 5 Institute of Pathology, Fudan University, Shanghai, 200032 China

**Keywords:** MHC-II molecules, Affinity prediction, Positional embedding

## Abstract

The prediction of major histocompatibility complex (MHC)-peptide binding affinity is an important branch in immune bioinformatics, especially helpful in accelerating the design of disease vaccines and immunity therapy. Although deep learning-based solutions have yielded promising results on MHC-II molecules in recent years, these methods ignored structure knowledge from each peptide when employing the deep neural network models. Each peptide sequence has its specific combination order, so it is worth considering adding the structural information of the peptide sequence to the deep model training. In this work, we use positional encoding to represent the structural information of peptide sequences and validly combine the positional encoding with existing models by different strategies. Experiments on three datasets show that the introduction of position-coding information can further improve the performance built upon the existing model. The idea of introducing positional encoding to this field can provide important reference significance for the optimization of the deep network structure in the future.

**Supplementary Information:**

The online version contains supplementary material available at 10.1186/s12864-023-09900-6.

## Introduction

T-cells present on their surface a specific receptor known as the T-cell receptor (TCR) that enables the recognition of antigens when they are displayed on the surface of antigen-presenting cells (APCs) bound to major histocompatibility complex (MHC) molecules [1, 2], which play a significant role in the adaptive immune response mediated by T cells [3, 4]. Due to the time-consuming and labor-intensive process of biochemical experiments [5, 6], the machine learning-based method of predicting MHC binding peptides has attracted more and more attention and has been used to optimize the selection of a small number of promising high-affinity binding peptides, which are further verified by biochemical experiments [7–9]. In general, there are two major classes of MHC molecules: MHC Class I (MHC-I) and MHC Class II (MHC-II) with subclasses in each of these two classes. MHC-I mainly has A, B, and C subclasses, while MHC-II mainly has DP, DQ, and DR subclasses encoded in the human leukocyte antigen (HLA) gene [10] and in the histocompatibility2 (H-2) gene of mouse [11]. Furthermore, note that different from MHC-I molecules consisting of one chain [12], each MHC-II molecule has two chains, $\alpha$ and $\beta$ [13, 14].

Most MHC-I peptide ligands have 9 residues, made from a single chain $\alpha$, and can promise better predicted results for these peptides that hold this size [15]. However, the MHC-II molecules’ peptide binding groove is open at both ends, allowing the peptide to extend beyond the binding groove (9-22 residues in length), although there is only a core of nine residues in the MHC-II binding groove [16, 17]. Therefore, peptide binding predictions for MHC-II molecules are of great challenge compared to those for MHC-I molecules. Therefore, how to design an effective algorithm framework to predict affinity peptides for MHC-II molecules has become a hot but difficult topic [18, 19]. To date, a variety of methods have been developed to predict the binding capacity of peptides to MHC-II [20–22], among which prediction of MHC-II peptide binding based on panspecificity is the most common and efficient computational solution [23, 24].

Early panspecific-based methods can be divided into various techniques, such as support vector machine (SVM) [25], motif matrix (MM) [26], artificial neural network (ANN) [27], and kernel-based methods [28]. Furthermore, NetMHCIIpan-4.0, an ANN-based method, exploited customized machine learning strategies to integrate different types of training data, resulting in happy performance and outperforming their competitors in that year [29]. Recently, pan-specific based methods began to be oriented toward deep learning (DL) [30]: PUFFIN used a deep residual network-based computational approach that quantifies uncertainty in peptide-MHC affinity prediction; The attention mechanism used by MHCAttnNet provided a heatmap over the peptide sequences [31]; And DeepSeaPanII was an end-to-end neural network model without the need for pre- or post-processing on input samples [32]. It is worth noting that the above DL-based methods only encode the text information of the peptide sequences and input them into the corresponding model, and do not take into account the structural information of the peptide sequence or its implicit presence in the network structure design [33].

**Fig. 1:**
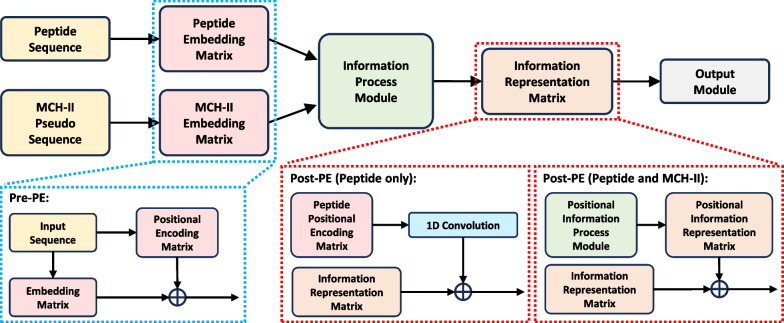
The architecture of our proposed method. The blue dashed box denotes the Pre-PE condition and the red dashed box denotes the Post-PE condition

Although some traditional methods utilize the structural information of the peptide sequences [34, 35], it is difficult to combine these deep learning algorithms properly. In recent research in the field of natural language processing (NLP) [36], positional encoding (PE) is used to encode the relative position of words in a sentence, allowing deep models to retain position information among words [37, 38]. Supplementing the structure information can effectively help the deep network to achieve better performance, especially those networks that are not sensitive to position information, such as the Transformer [39]. Apart from this, [40] showed that the structural information was crucial for modular reinforcement learning, substantially outperforming prior state-of-the-art methods on multi-task learning [41, 42]. Therefore, in the MHC-II affinity peptide prediction task, the introduction of position encoding can be expected to further improve the performance of the training deep-learning model, especially when the internal position of each peptide sequence is completely determined [43].

In this paper, the proposed algorithm is based on DeepMHCII, the current state-of-the-art DL-based algorithm [44]^1^, to validate our proposed strategy. To study the effectiveness of introducing positional encoding, this paper discusses the placement of positional encoding in different positions and the use of different encoding schemes. To intuitively compare the performance of different positional encoding-adding strategies, the same datasets and evaluation as the DeepMHCII algorithm are used, such as 5-fold cross-validation, independent testing set verification, and binding core prediction. Experimental results show that the introduction of positional encoding information can further improve the performance of the DeepMHCII model. We believe that introducing position encoding into this task can provide an important reference for future model optimization.

## Methods

### Preliminary

Consider two primary sequences: a peptide sequence denoted by *P* and an MHC-II molecule sequence denoted by *Q*. Both sequences consist of the 20 standard amino acids. Our objective is to establish a regression model that predicts the binding affinity $\hat{ z } \in [0, 1]$ when given a specific pair of *P* and *Q*. The binding affinity between *P* and *Q* is primarily influenced by two factors:

- The peptide’s binding core: The principal segment actively participates in interactions with the MHC-II molecule.
- The MHC-II molecule’s binding groove: A region marked by its nine specialized pockets, which is paramount for the peptide’s accommodation.

In addition, it should be noted that peptide flanking residues (PFRs) play a role. Although these residues reside outside the core binding groove, research has shown their significant impact. PFRs not only influence the binding affinity of the peptide, but also play a role in enhancing both peptide processing and T cell activation [45]. To facilitate our study and in alignment with prior research settings, we focus on a 34-residue pseudo sequence derived from *Q*. This representation of MHC-II molecules is an amalgamation of two parts: 15 amino acid residues from the $\alpha$ chain and 19 from the $\beta$ chain of MHC-II. The extraction of these residues is based on their presence in the MHC-II peptide complexes found in the Protein Data Bank (PDB) [46].

### Overview of DL-based MHC-II binding prediction

The burgeoning field of immunological research has turned to Deep Learning (DL) methodologies, especially panspecific methods, to offer granular insights [47]. These methods can be distilled into a structured deep framework as shown in Fig. 1. Based on this, existing frameworks employ an embedding layer dedicated to encoding peptide sequences into an embedding matrix, $\textbf{X} \in \mathbb {R}^{L_{peptide} \times d}$. Currently, another separate embedding layer focuses on translating the pseudo-sequences of MHC-II into a unified embedding matrix, $\textbf{Y} \in \mathbb {R}^{L_{pseudo} \times d}$. After initial encoding, both embedding matrices undergo a transformation mediated by the **information process module**. In doing this, sophisticated structures are tailored to extract the underlying **Information Representation Matrix**, revealing the dynamic interplay between peptides and MHC-II molecules. Drawing the process to a close, the framework takes advantage of an output layer. Its primary objective is dual: to calculate the binding affinity, denoted by $\hat{ z }$, and to discern the predictive scores associated with potential binding cores of nine lengths.

The purpose of this paper is to delve deeper into the architectural intricacies of this framework. Emphasis will be laid on the integration of structural data, with a dedicated segment elucidating the strategic application of positional encoding to bolster the prediction accuracy. When considering positional encoding (PE) strategies for peptide sequences in the context of machine learning, especially for tasks like predicting the binding affinity between peptides and MHC-II molecules, various strategies can be formulated by combining different aspects of positional encoding. These strategies could include variations in the position of the addition of encoding, the method of encoding, and the use of positional peptide encoding alone. Let us explore what each of these aspects means and how they can be combined.

### How to implement the positional encoding

In this section, we explore the computation of positional encoding for the peptide sequence and the MHC-II pseudo-sequence. Let us consider a peptide sequence, *P*, and an MHC-II pseudo-sequence, *Q*. These sequences are mapped to their corresponding positional encodings, $\mathbf {X_{PE}} \in \mathbb {R}^{L \times d}$ and $\mathbf {Y_{PE}} \in \mathbb {R}^{34 \times d}$, representing the positional encoding matrices for *P* and *Q*, respectively. The MHC-II pseudo-sequence *Q* is a 34-length sequence extracted from the entire MHC-II molecule sequence. The positional encoding can be defined as:

1 $$\begin{aligned} \mathbf{X}_{\mathbf{PE}} = (x_1, x_2, \ldots , x_L)^\top \in \mathbb{R}^{L \times d}, \quad \mathbf {Y}_{\mathbf{PE}} = (y_1, y_2, \ldots , y_{34})^\top \in \mathbb {R}^{34 \times d}, \end{aligned}$$

where $x_i \in \mathbb {R}^{d}$ denotes the positional encoding vector for the *i*-th residue of the peptide sequence, and $y_j \in \mathbb {R}^{d}$ represents the positional encoding vector for the *j*-th residue of the MHC-II pseudo-sequence. Here, *L* signifies the length of the input peptide sequence.

**Fig. 2:**
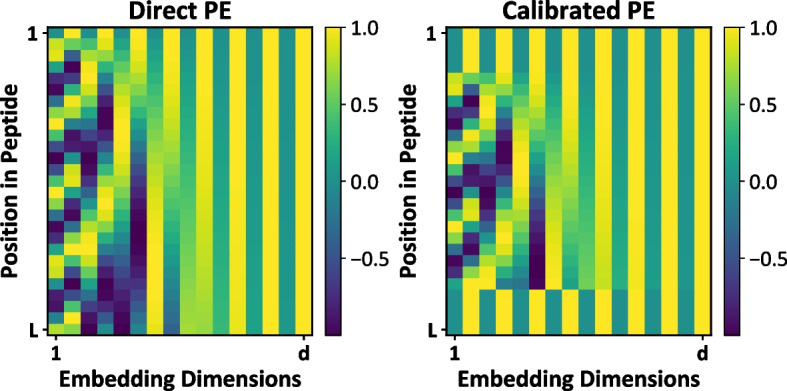
Illustration of two different positional encodings: Direct PE and Calibrated PE

Due to the peptide-binding groove alignment on MHC-II molecules being open at both ends [48], which allows the peptide to extend beyond the binding groove (9-22 residues in length), practical sequences of equal length are achieved by zero padding at both ends of the peptide sequence. Thus, amino acids at these zero positions will produce invalid encoding which has been considered by the traditional peptide embedding process. Therefore, this article proposed a new positional encoding strategy for the encoding of the peptide sequence, Calibrated PE, based on Direct PE, as shown in Fig. 2. The positional encoding of a sequence P when using calibrated PE can be rewritten as follows:

2 $$\begin{aligned} \mathbf {X_{C-PE}}=(x_0,...,x_0,x_1,...x_{L'},x_0,...,x_0)^T \in \mathbb {R}^{L \times d}, \end{aligned}$$

where $L'$ denotes the truth length of the peptide sequence. For computing positional encoding, a fixed sinusoidal relative PE method that is derived from sinusoidal functions and fixed during the model training [49]. For example, consider the dimension $2k, (x_2, x_4,..., x_{2k},...)$ and the dimension $2k+1, (x_1, x_3,..., x_{2k+1},...)$ of a peptide-encoded PE, respectively:

3 $$\begin{aligned} \text {PE}(pos, 2k)={} & {} sin\left( \frac{pos}{1000^{2k/d}}\right) \nonumber \\ \text {PE}(pos, 2k+1)={} & {} cos\left( \frac{pos}{1000^{2k+1/d}}\right) \end{aligned}$$

### Where to add the positional encoding

The next thing to consider is where to add the positional encoding information. Positional encoding information can be inserted into the pre (pre-PE) or post (post-PE) information process module according to Fig. 1, respectively. Here, the information process module is the binding interaction convolutional layer (BICL) proposed by DeepMHCII. Briefly, BICL generates different kernels for each MHC-II molecule and performs a convolution operation on the peptide embedding matrix. On the other hand, we also consider whether the positional encoding is applied exclusively to the peptide sequence, which may highlight the importance of the peptide’s position in binding without affecting the encoding of the MHC-II molecule, or to both the peptide and MHC-II sequences, allowing the model to learn the relative positions of both in the binding interaction.

As for Pre-PE, just simply add the position-encoding information matrix to the amino acid embedding matrix to make the trained model contain position-encoding information, shown in the blue dashed box of Fig. 1. Let $\mathbf {X_{Emb}} \in \mathbb {R}^{L \times d}$ and $\mathbf {Y_{Emb}} \in \mathbb {R}^{34 \times d}$, representing the embedding matrices for *P* and *Q*, respectively. Thus, the combined embedding matrices which will undergo a transformation mediated by the Information Process Module can be given as follows:

4 $$\begin{aligned} \textbf{X} = \mathbf {X_{Emb}} + \mathbf {X_{PE}} (or \ \mathbf {X_{C-PE}}) \in \mathbb {R}^{L \times d}, \quad \textbf{Y} = \mathbf {Y_{Emb}} + \mathbf {Y_{PE}} \in \mathbb {R}^{34 \times d}. \end{aligned}$$

When it comes to Post-PE (the red dashed box of Fig. 1), things are going to get a little more complicated. As can be seen from DeepMHCII framework, the peptide sequence information representation matrix, denoted as the output of BICL can be written as:

5 $$\begin{aligned} C_{Emb}=f\left( f\left( W^k \textbf{Y}\right) \textbf{X}+b^k\right) , \end{aligned}$$

Where $W^k$ is the weight matrix, $b^k$ is the bias, *f* is the activation function, and *k* denotes different kernel sizes. Considering the effect of both the binding core and PFRs, BICL used four different kernel sizes ($s^k$): 9, 11, 13, and 15. For each kernel size, there is a different number of kernels ($h^k$). In the peptide-only strategy, a 1D convolution layer is used to learn the positional information representation matrix and add this to the output of BICL. To satisfy the size with the output of BICL, the kernels ($W_{PE-O}^k$) of convolution hold the size of $h^k \times d \times s^k$ and the output of this layer can be described as follows:

6 $$\begin{aligned} C_{PE}=f\left( W_{PE-O}^k \cdot \textbf{X}_{PE}+b_{PE-O}^k\right) , \end{aligned}$$

where $b_{PE-O}^k$ is the bias, *f* is the activation function, and *k* denotes different kernel sizes. In both considering the peptide and the MHC-II strategy, the same structure of BICL was employed and the interaction between $\textbf{X}_{PE}$ and $\textbf{Y}_{PE}$ can be given as follows:

7 $$\begin{aligned} C_{PE}=f\left( f\left( W_{PE-T}^k \textbf{Y}_{PE}\right) \cdot \textbf{X}_{PE}+b_{PE-T}^k\right) , \end{aligned}$$

where $W_{PE-T}^k$ with the size of $h^k \times s^k \times 34$ to generate the kernels and $b_{PE-T}^k$ is the bias. Same as the Pre-PE condition, the combined information representation matrix can be given as follows:

8 $$\begin{aligned} C=C_{Emb}+C_{PE}, \end{aligned}$$

### Datasets

Three available benchmark datasets are used to train and evaluate our proposed method:

- BC2015: a binding core benchmark, which was used to evaluate the performance of NetMHCIIpan3.2^2^ in identifying the binding core of an MHC-II peptide complex. BC2015 consists of 51 complexes from PDB.
- BD2016: It contains 134,281 data points on MHC-peptide binding affinities for 80 different MHC-II molecules, including 36 HLA-DR, 27 HLA-DQ, 9 HLA-DP and 8 H-2 molecules. BD2016 already provides a 5-fold cross-validation (5-fold CV) split that groups peptides with common motifs into the same fold.
- ID2017: an independent test dataset in DeepMHCII, ID2017, by removing data points that overlapped with BD2016 and retained MHC-II molecules with more than 50 peptides for robust performance evaluation. There are 10 HLA-DB molecules with 857 peptides in practice.

The following experiments will be conducted to validate the performance of our method: (i) the performance comparison among different PE-adding strategies on ID2017; (ii) the performance of different PE-adding strategies by 5-fold CV over BD2016; (iii) visualization of the binding motifs of MHC-II molecules obtained by each model as sequence logos; (iv) predict the binding core over BC2015.

We have set the minimization of the mean square error as our primary goal in training. To achieve this, we have implemented an ensemble learning strategy, wherein we trained *T* distinct models, each initialized with unique random weights. The final prediction is derived by calculating the mean of the predictions of all *T* models.

The area under the receiver operating characteristic curve (AUC) for each MHC-II molecule and the average AUC were reported. The Pearson correlation coefficient (PCC) was calculated to examine the linear relationship between the predicted binding affinity. Spearman rank correlation coefficient (SRCC) measured the monotonic relationship based on ranks. Furthermore, mean square error (MSE) was used to provide a measure of the average prediction error of the proposed strategy.

## Results

### Experimental settings

In this paper, the following hyperparameter values can be found, which are the same as DeepMHCII: $d = 16$. The number of kernels $h^k$ with kernel sizes $s^k$ of 9, 11, 13, and 15 was 256, 128, 64, and 64, respectively. *f* was ReLU. While training, the batch size was 128, the number of epochs was 20 and the optimizer was Adaelta [50] with a learning rate of 0.9 and weight decay of 1e-4. *T* (number of trained models) was 20. Apart from that, this paper discussed 8 types of combined PE-adding strategies: 2 PE-adding positions (where), 2 PE encoding methods (how), and consider whether to use peptide-only PE. Each strategy would have its advantages and could be tested empirically to see which yields the associated accurate predictions.

### Comparison of different pe-adding strategies on ID2017

Table 1 offers a detailed analysis of the performance (AUC) on the ID2017 independent test set when different positional encoding (PE) strategies are applied to the baseline DeepMHCII model. The tables elucidate that Calibrated PE, particularly the Pre-PE(T) strategy, outperforms other configurations with an impressive AUC of 0.777, marking an enhancement over the baseline performance of DeepMHCII, which has an average AUC of 0.770. Examining individual allele performance further, the Calibrated PE method demonstrates a clear advantage. For instance, allele DRB1*0301 shows a significant increase from an AUC of 0.629 with DeepMHCII to 0.676 with Calibrated Pre-PE(T). Similarly, allele DRB1*0701’s AUC escalates to 0.845 with Calibrated Post-PE(T), compared to 0.814 with the baseline. However, not all alleles react equally to the addition of PEs. Allele DRB1*0401, for example, maintains a higher AUC with the baseline DeepMHCII at 0.863, compared to 0.790 with Calibrated Pre-PE(T). Across the alleles, Calibrated PE’s average AUC exhibits a notable increment compared to Direct PE’s average AUC. More importantly, the same conclusion can be drawn from the results in Tables A1 and A2. These observations corroborate the inference that calibrated PE, especially the Pre-PE(T) strategy, is generally more beneficial than direct PE in improving the prediction accuracy of the DeepMHCII model on the ID2017 dataset.

**Table 1:** Performance (AUC) of using different positional encoding conditions and DeepMHCII on ID2017. ’O’ denotes using peptide-PE only, and ’T’ denotes using peptide-PE and MHC-II-PE together

Allele	DeepMHCII	Calibrated PE				Direct PE			
		Pre (O)	Pre (T)	Post (O)	Post (T)	Pre (O)	Pre (T)	Post (O)	Post (T)
DRB1*0101	0.882	0.868	0.873	**0.885**	0.871	0.883	0.883	0.875	0.877
DRB1*0301	0.629	0.602	**0.676**	0.622	0.632	0.628	0.620	0.629	0.610
DRB1*0401	**0.863**	0.813	0.790	0.854	0.814	0.807	0.778	0.810	0.816
DRB1*0701	0.814	0.792	**0.845**	0.802	0.823	0.812	0.821	0.825	0.821
DRB1*0901	0.889	0.875	0.844	**0.893**	0.859	0.856	0.844	0.841	0.842
DRB1*1101	0.657	0.642	0.649	**0.658**	0.648	0.652	0.641	0.628	0.633
DRB1*1202	0.788	0.758	**0.811**	0.763	0.725	0.788	0.733	0.716	0.735
DRB1*1301	0.615	0.566	**0.736**	0.636	0.660	0.517	0.651	0.643	0.572
DRB1*1501	0.799	0.798	0.821	0.807	**0.823**	0.810	0.798	0.817	0.806
DRB1*1502	**0.764**	0.752	0.728	0.734	0.700	0.726	0.720	0.679	0.687
Average	0.770	0.747	**0.777**	0.765	0.755	0.748	0.749	0.746	0.740

### Comparison of different PE-adding strategies on BD2016

**Fig. 3:**
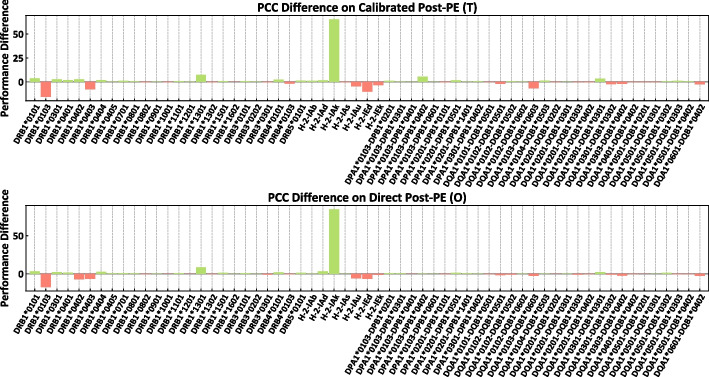
PCC difference in Calibrated Post-PE (T) and Direct Post-PE (O) surpasses that in DeepMHCII

Table 2 delineates the performance metrics of various positional encoding (PE) strategies in comparison to the baseline DeepMHCII on the BD2016 dataset. A careful examination of the figures reveals that Post-PE configurations generally surpass Pre-PE strategies across all measured criteria. This finding contrasts with the observations from the ID2017 dataset, where the Calibrated Pre-PE(T) strategy was notably effective. In particular, the Direct Post-PE(O) approach outperforms other strategies with the highest AUC of 0.857, which is marginally better than the baseline DeepMHCII’s AUC of 0.856. Similarly, this strategy achieved the top Pearson Correlation Coefficient (PCC) of 0.694, slightly improving upon the baseline’s 0.691. This trend continues with Spearman’s Rank Correlation Coefficient (SRCC), where the Direct Post-PE(O) achieves an SRCC of 0.685, approaching the highest value in the table, 0.687, as seen with Calibrated Post-PE(T). Furthermore, the Mean Squared Error (MSE) metric also supports the superiority of Post-PE methods. Both Calibrated Post-PE(T) and Direct Post-PE(O) strategies share the lowest MSE of 0.0299, indicating a statistically significant reduction from the DeepMHCII baseline of 0.0308. These results highlight that, for the BD2016 dataset, the application of Post-PE, especially the Direct Post-PE(O) strategy, is particularly beneficial, surpassing the pre-encoding strategies and improving upon the baseline DeepMHCII model across multiple performance metrics. Detailed results for BD2016 are shown in Tables A3, A4 and A5. Besides, we further showed the PCC difference in Calibrated Post-PE (T) and Direct Post-PE (O) surpasses that in DeepMHCII (Fig. 3).

**Table 2:** Performance of using different positional encoding conditions and DeepMHCII on BD2016

Allele	DeepMHCII	Calibrated PE				Direct PE			
		Pre (O)	Pre (T)	Post (O)	Post (T)	Pre (O)	Pre (T)	Post (O)	Post (T)
AUC	0.856	0.844	0.820	0.855	0.856	0.843	0.820	**0.857** $\uparrow$	0.855
PCC	0.691	0.679	0.634	0.689	0.693$\uparrow$	0.675	0.633	**0.694** $\uparrow$	0.692$\uparrow$
SRCC	0.682	0.672	0.630	0.681	**0.687** $\uparrow$	0.666	0.626	0.685$\uparrow$	0.686$\uparrow$
MSE	0.0308	0.0301$\downarrow$	0.0337	0.0312	**0.0299** $\downarrow$	0.0315	0.0341	**0.0299** $\downarrow$	0.0301$\downarrow$

### Binding core prediction and sequence logos

**Fig. 4:**
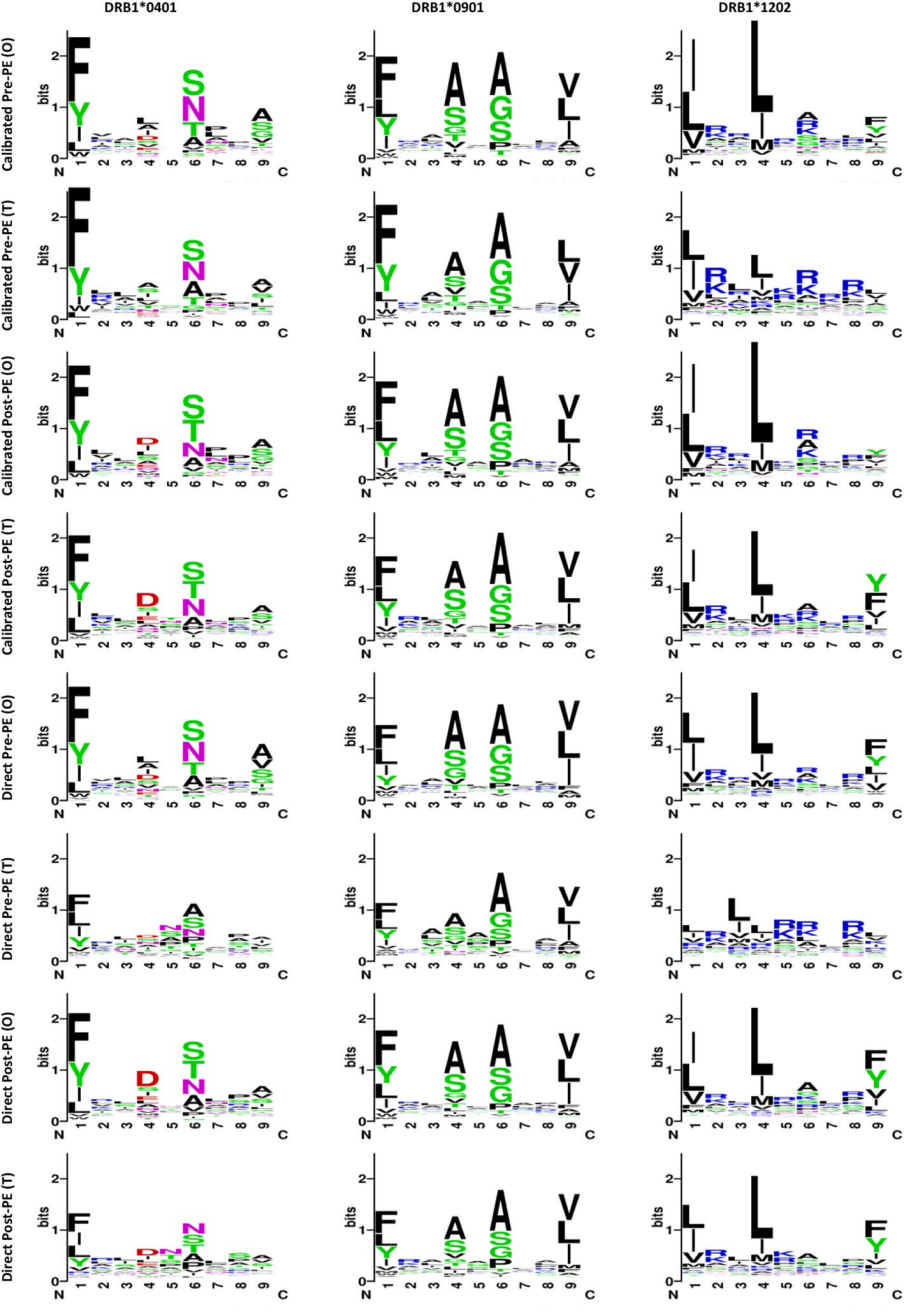
Sequence logos by using different positional encoding

Furthermore, we have graphically represented the binding motifs of MHC-II molecules derived from each PE-adding strategy in the form of sequence logos, which are accessible at the WebLogo portal^3^ [51, 52]. For illustrative purposes, we selected three MHC-II molecules-DRB10401, DRB10901, and DRB1*1202-from the ID2017 set and subjected them to random testing. Figure 4 displays the sequence logos corresponding to these MHC-II molecules as influenced by different encoding strategies. On the sequence logos, the x-axis encompasses positions 1 through 9 (referred to as pockets), where the overall height at each position is indicative of the relative informational significance attributed to that specific site within the motif. Concurrently, the stature of individual letters within each position correlates to the prevalence of the respective amino acid at that site. Typically, pockets 1, 4, 6, and 9 constitute the four main anchor positions, considered critical for the binding affinity of peptides [53]. The findings suggest that all strategies except for Direct Post-PE(O) exhibited substantial promise and conferred valuable insights pertinent to peptide binding. Upon evaluating the prediction accuracy of the binding core over the BC2015 dataset, the performance of Calibrated PE was observed to eclipse that of Direct PE, as evidenced by the data articulated in Tables A6 and A7.

## Discussion

In this study, we introduced a novel approach to integrate structural information to enhance the predictions of binding affinity of MHC-II molecule using the DeepMHCII framework. The results from our proposed methodology indicate a noticeable improvement in DeepMHCII’s performance, affirming the utility of positional encoding (PE) strategies in this domain. The comprehensive analysis provided herein lays a foundation for empirical understanding and sets a precedent for future investigative pursuits. Intriguingly, our findings underscore the variability in performance outcomes contingent upon the dataset and evaluative measures employed, thereby suggesting a necessity for meticulously tailored positional encoding techniques to accommodate different research requirements.

While our results are promising, they also reveal the heterogeneity of performance across different datasets and evaluation metrics, suggesting that a one-size-fits-all approach to positional encoding may not be viable. Instead, there is a compelling need for the careful design of positional information that aligns with specific tasks and datasets. Moreover, the landscape of positional encoding methodologies extends beyond the scope of our current work, with avenues such as learnable PEs presenting opportunities for further exploration. These adaptive encoding methods could potentially reveal more nuanced structural relationships within the binding affinities of MHC-II molecules.

Potential applications of this research are vast, including the development of more accurate predictive models for vaccine design, where understanding peptide-MHC-II interactions is crucial. Such models could substantially expedite the identification of potent epitopes, thereby bolstering the development of peptide-based vaccines and therapeutics. However, the limitations must be acknowledged. One such constraint is the reliance on available structural data, which may not fully capture the dynamic nature of peptide-MHC interactions. Future work could aim to incorporate three-dimensional spatial structures, potentially offering a more holistic view of the binding process. This could be achieved through the integration of molecular dynamics simulations or the application of advanced imaging techniques, further enhancing the predictive capabilities of deep learning models in this field. Ultimately, our work serves as a stepping stone toward the realization of deep learning methodologies that not only utilize positional information, but also encapsulate the rich structural intricacies inherent to biological processes. This could pave the way for a new era of bioinformatic tools capable of tackling complex biological predictions with greater accuracy and efficiency.

### Supplementary Information

**Additional file 1.**

## Data Availability

All data generated or analyzed during this study are included in this published article; our manuscript does not contain any data, as no data set was generated or analyzed during the study.

## Footnotes

1 https://github.com/yourh/DeepMHCII

2 http://www.cbs.dtu.dk/suppl/immunology/NetMHCIIpan3.2

3 https://weblogo.berkeley.edu/
