# Supplementary material for: Structure-aware deep model for MHC-II peptide binding affinity prediction
Source: BMC Genomics. 2024 Jan 30;25:127. doi: 10.1186/s12864-023-09900-6 (PMC10826266; doi:10.1186/s12864-023-09900-6)
Supplement: Supplementary file 1 — Additional file 1. [file 12864_2023_9900_MOESM1_ESM.pdf]

Structure-aware deep model for MHC-II peptide binding affinity prediction

Ying Yu[1†], Lipeng Zu[2†], Jiaye Jiang[1], Yafang Wu[1], Yinglin Wang[1], Midie Xu[3,4,5*], Qing Liu[1*]

[1*]School of Health Science and Engineering, University of Shanghai for Science and Technology, Shanghai, 200093, China.
[2]Department of Computer Science, Florida State University, Tallahassee, 32306, United States.
[3]Department of Pathology, Fudan University Shanghai Cancer Center, Shanghai, 200032, China.
[4]Department of Medical Oncology, Shanghai Medical College, Fudan University, Shanghai, 200032, China.
[5]Institute of Pathology, Fudan University, Shanghai, 200032, China.

*Corresponding author(s). E-mail(s): xumd27202003@sina.com; liuq@usst.edu.cn;
Contributing authors: yuying2000@yeah.net; lz23b@fsu.edu; 760346922@qq.com; 2570463373@qq.com; 1034300874@qq.com;
†These authors contributed equally to this work.

Abstract

The prediction of major histocompatibility complex (MHC)-peptide binding affinity is an important branch in immune bioinformatics, especially helpful in accelerating the design of disease vaccines and immunity therapy. Although deep learning-based solutions have yielded promising results on MHC-II molecules in recent years, these methods ignored structure knowledge from each peptide When employing the deep neural network models. Each peptide sequence has its specific combination order, so it is worth considering adding the structural information of the peptide sequence to the training of the deep model. In this work, we use positional encoding to represent the structural information of peptide sequences and validly combine the positional encoding with existing models by different strategies. Experiments on three datasets show that the introduction of position coding information can further improve the performance built upon the existing model. The idea of introducing positional encoding to this field can

provide important reference significance for the optimization of deep network structure in the future.

Keywords: MHC-II Molecules, Affinity Prediction, Positional Embedding

Appendix A

Table A1 Performance (PCC) of using different positional encoding conditions on the independent testing set.

Allele	Calibrated PE				Direct PE			
	Pre (O)	Pre (T)	Post (O)	Post (T)	Pre (O)	Pre (T)	Post (O)	Post (T)
DRB1*0101	0.500	0.504	**0.533**	0.501	0.494	0.516	0.490	0.504
DRB1*0301	0.190	**0.271**	0.213	0.230	0.234	0.183	0.218	0.203
DRB1*0401	0.525	0.500	**0.597**	0.525	0.524	0.482	0.522	0.530
DRB1*0701	0.408	**0.530**	0.440	0.474	0.448	0.473	0.467	0.476
DRB1*0901	**0.637**	0.635	0.611	0.587	0.600	0.614	0.551	0.562
DRB1*1101	0.240	0.264	**0.270**	0.241	0.251	0.240	0.214	0.223
DRB1*1202	0.355	**0.404**	0.353	0.281	0.399	0.327	0.286	0.296
DRB1*1301	0.099	**0.241**	0.162	0.185	0.046	0.123	0.161	0.131
DRB1*1501	0.363	**0.409**	0.369	0.387	0.377	0.374	0.386	0.375
DRB1*1502	**0.413**	0.392	0.343	0.300	0.404	0.372	0.227	0.264
Average	0.373	**0.415**	0.389	0.371	0.378	0.370	0.352	0.356

Table A2 Performance (SRCC) of using different positional encoding conditions on the independent testing set.

Allele	Calibrated PE				Direct PE			
	Pre (O)	Pre (T)	Post (O)	Post (T)	Pre (O)	Pre (T)	Post (O)	Post (T)
DRB1*0101	0.500	0.506	**0.523**	0.505	0.521	0.521	0.509	0.513
DRB1*0301	0.172	**0.297**	0.206	0.222	0.216	0.202	0.217	0.185
DRB1*0401	0.521	0.482	**0.589**	0.522	0.510	0.463	0.515	0.524
DRB1*0701	0.429	**0.506**	0.443	0.473	0.457	0.471	0.477	0.471
DRB1*0901	0.579	0.532	**0.607**	0.555	0.551	0.532	0.526	0.528
DRB1*1101	0.208	0.219	**0.232**	0.217	0.223	0.206	0.187	0.195
DRB1*1202	0.348	**0.419**	0.355	0.304	0.388	0.314	0.291	0.317
DRB1*1301	0.087	**0.311**	0.179	0.210	0.022	0.199	0.188	0.095
DRB1*1501	0.375	0.403	0.386	**0.406**	0.390	0.375	0.399	0.385
DRB1*1502	**0.374**	0.338	0.346	0.296	0.335	0.327	0.265	0.277
Average	0.359	**0.401**	0.387	0.371	0.361	0.361	0.358	0.349

Appendix B

Table A3 Detail Performance (AUC) of using different positional encoding conditions on BD2016.

Allele	Calibrated PE				Direct PE			
	Pre (O)	Pre (T)	Post (O)	Post (T)	Pre (O)	Pre (T)	Post (O)	Post (T)
DRB1*0101	0.844	0.837	0.836	**0.852**	0.842	0.835	0.850	0.851
DRB1*0103	0.566	0.539	**0.678**	0.526	0.533	0.572	0.586	0.586
DRB1*0301	0.822	0.803	0.832	**0.836**	0.821	0.799	**0.836**	0.834
DRB1*0401	0.816	0.802	0.816	**0.822**	0.815	0.799	0.820	0.818
DRB1*0402	0.652	**0.707**	0.672	0.695	0.678	0.667	0.659	0.646
DRB1*0403	0.868	0.851	**0.873**	0.802	0.857	0.851	0.808	0.821
DRB1*0404	0.824	0.813	0.817	**0.826**	0.821	0.809	0.825	0.823
DRB1*0405	0.836	0.822	0.843	0.844	0.836	0.820	**0.846**	0.843
DRB1*0701	0.878	0.869	0.887	**0.889**	0.877	0.869	0.888	0.888
DRB1*0801	0.847	0.822	0.857	**0.860**	0.847	0.823	**0.860**	0.857
DRB1*0802	0.836	0.825	0.838	**0.839**	**0.839**	0.825	**0.839**	0.838
DRB1*0901	0.835	0.828	**0.840**	0.838	0.835	0.826	**0.840**	0.839
DRB1*1001	**0.922**	0.914	0.917	0.920	0.921	0.912	0.920	0.916
DRB1*1101	0.871	0.856	0.872	0.875	0.872	0.853	**0.876**	0.872
DRB1*1201	0.879	0.852	**0.888**	0.887	0.878	0.851	0.887	0.883
DRB1*1301	**0.864**	0.839	0.819	0.841	0.863	0.842	0.843	0.838
DRB1*1302	0.885	0.869	**0.887**	0.883	0.882	0.862	0.886	0.883
DRB1*1501	0.844	0.833	0.846	0.849	0.845	0.829	**0.850**	**0.850**
DRB1*1602	0.888	0.881	**0.893**	0.892	0.890	0.880	0.892	0.892
DRB3*0101	0.901	0.888	0.903	0.905	0.897	0.884	**0.906**	0.905
DRB3*0202	0.877	0.852	**0.889**	0.887	0.872	0.852	0.888	**0.889**
DRB3*0301	0.837	0.817	**0.851**	0.850	0.830	0.816	0.848	0.846
DRB4*0101	0.826	0.810	0.842	**0.847**	0.828	0.810	**0.847**	0.846
DRB4*0103	0.826	0.828	0.831	0.835	0.826	0.828	**0.836**	0.834
DRB5*0101	0.863	0.855	0.864	**0.868**	0.861	0.852	**0.868**	**0.868**
H-2-IAb	0.883	0.860	**0.899**	**0.899**	0.881	0.863	0.895	0.895
H-2-IAd	0.841	0.806	0.835	0.847	0.836	0.805	**0.857**	0.851
H-2-IAk	0.390	0.374	0.592	0.773	0.428	0.430	**0.806**	0.676
H-2-IAs	0.807	0.746	0.798	0.831	0.805	0.755	**0.836**	0.818
H-2-IAu	0.834	0.790	**0.852**	0.789	0.799	0.779	0.798	0.845
H-2-IEd	0.736	0.685	0.737	0.700	**0.766**	0.691	0.730	0.716
H-2-IEk	**0.862**	0.794	0.805	0.797	0.810	0.780	0.796	0.840
DPA1*0103-DPB1*0201	0.919	0.899	0.917	**0.930**	0.920	0.901	0.926	0.927
DPA1*0103-DPB1*0301	0.900	0.885	0.906	0.911	0.900	0.886	0.911	**0.913**
DPA1*0103-DPB1*0401	0.934	0.926	**0.942**	0.941	0.935	0.925	0.941	0.940
DPA1*0103-DPB1*0402	0.799	0.710	0.802	**0.821**	0.793	0.722	0.802	0.799
DPA1*0103-DPB1*0601	0.995	0.994	0.994	0.994	**0.996**	0.994	0.995	0.995
DPA1*0201-DPB1*0101	0.903	0.894	**0.911**	0.909	0.902	0.893	0.910	**0.911**
DPA1*0201-DPB1*0501	0.914	0.902	0.914	**0.921**	0.914	0.902	**0.921**	**0.921**
DPA1*0201-DPB1*1401	0.942	0.930	0.944	0.946	0.941	0.931	**0.947**	**0.947**
DPA1*0301-DPB1*0402	0.904	0.893	**0.911**	0.910	0.900	0.894	0.908	0.908
DQA1*0101-DQB1*0501	0.900	0.871	**0.915**	0.911	0.898	0.871	0.912	0.912
DQA1*0102-DQB1*0501	**0.842**	0.777	0.820	0.818	0.836	0.764	0.822	0.818
DQA1*0102-DQB1*0502	0.845	0.781	0.855	**0.865**	0.845	0.791	0.859	0.850
DQA1*0102-DQB1*0602	0.886	0.870	0.904	0.904	0.886	0.869	0.906	**0.907**
DQA1*0103-DQB1*0603	0.865	0.818	**0.872**	0.851	0.865	0.834	0.863	0.862
DQA1*0104-DQB1*0503	0.789	0.727	0.838	**0.848**	0.790	0.720	0.840	0.837
DQA1*0201-DQB1*0202	0.826	0.784	0.846	0.843	0.827	0.786	**0.847**	0.841
DQA1*0201-DQB1*0301	0.856	0.840	0.863	0.867	0.858	0.837	0.869	**0.872**
DQA1*0201-DQB1*0303	0.887	0.880	**0.902**	0.898	0.880	0.882	0.897	0.893
DQA1*0201-DQB1*0402	0.835	0.802	**0.874**	0.868	0.839	0.800	0.872	0.870
DQA1*0301-DQB1*0301	0.814	0.791	0.816	0.836	0.829	0.785	0.825	**0.842**
DQA1*0301-DQB1*0302	0.832	0.808	**0.845**	0.837	0.830	0.803	**0.845**	0.843
DQA1*0303-DQB1*0402	0.826	0.724	**0.855**	0.840	0.825	0.710	0.835	0.835
DQA1*0401-DQB1*0402	**0.883**	0.863	0.872	0.880	0.882	0.867	0.879	0.877
DQA1*0501-DQB1*0201	0.877	0.859	**0.883**	0.881	0.878	0.860	0.882	0.882
DQA1*0501-DQB1*0301	0.919	0.903	**0.927**	0.924	0.914	0.899	**0.927**	0.926
DQA1*0501-DQB1*0302	0.837	0.805	0.843	0.847	0.834	0.815	**0.849**	0.848
DQA1*0501-DQB1*0303	0.879	0.866	0.883	**0.890**	0.874	0.869	0.883	0.887
DQA1*0501-DQB1*0402	0.853	0.813	0.864	**0.868**	0.848	0.812	0.866	**0.868**
DQA1*0601-DQB1*0402	0.790	0.712	**0.842**	0.834	0.790	0.708	0.835	**0.842**
Average	0.844	0.820	0.855	0.856	0.843	0.820	**0.857**	0.855

Table A4 Detail Performance (PCC) of using different positional encoding conditions on BD2016.

Allele	Calibrated PE				Direct PE			
	Pre (O)	Pre (T)	Post (O)	Post (T)	Pre (O)	Pre (T)	Post (O)	Post (T)
DRB1*0101	0.698	0.684	0.681	**0.713**	0.693	0.675	0.710	0.711
DRB1*0103	0.258	0.240	**0.278**	0.183	0.220	0.232	0.180	0.158
DRB1*0301	0.624	0.592	0.638	**0.655**	0.621	0.586	0.653	0.652
DRB1*0401	0.627	0.598	0.625	**0.641**	0.621	0.591	0.640	0.636
DRB1*0402	0.450	0.519	0.501	**0.525**	0.509	0.504	0.474	0.472
DRB1*0403	**0.758**	0.658	0.699	0.650	0.735	0.677	0.659	0.666
DRB1*0404	0.671	0.650	0.659	0.676	0.666	0.645	**0.680**	0.673
DRB1*0405	0.671	0.645	0.680	0.688	0.669	0.637	**0.689**	0.688
DRB1*0701	0.748	0.729	0.758	**0.768**	0.744	0.726	0.766	0.765
DRB1*0801	0.678	0.634	0.684	**0.698**	0.671	0.628	0.697	0.696
DRB1*0802	0.662	0.640	0.664	0.672	0.668	0.642	0.673	**0.674**
DRB1*0901	0.669	0.657	0.674	0.680	0.669	0.657	**0.681**	0.678
DRB1*1001	0.777	0.755	0.778	0.778	0.776	0.748	**0.780**	0.779
DRB1*1101	0.745	0.712	0.747	**0.752**	0.745	0.706	**0.752**	0.749
DRB1*1201	0.753	0.711	0.766	**0.770**	0.753	0.710	0.767	0.764
DRB1*1301	**0.689**	0.643	0.605	0.658	0.680	0.643	0.663	0.653
DRB1*1302	0.761	0.724	0.766	0.759	0.759	0.718	**0.762**	0.760
DRB1*1501	0.688	0.657	0.694	0.699	0.686	0.648	**0.701**	**0.701**
DRB1*1602	0.788	0.763	**0.791**	0.787	0.786	0.759	0.788	0.788
DRB3*0101	0.801	0.786	0.806	**0.811**	0.797	0.780	**0.811**	0.810
DRB3*0202	0.800	0.777	0.813	**0.814**	0.799	0.775	0.813	0.812
DRB3*0301	0.686	0.623	**0.709**	0.702	0.659	0.617	0.698	0.697
DRB4*0101	0.653	0.621	0.683	**0.697**	0.651	0.617	0.695	0.695
DRB4*0103	0.590	0.594	0.592	0.601	0.583	0.590	**0.608**	0.600
DRB5*0101	0.734	0.721	0.736	**0.745**	0.729	0.713	**0.745**	0.743
H-2-IAb	0.710	0.674	0.729	**0.736**	0.706	0.677	0.730	0.726
H-2-IAd	0.678	0.644	0.672	0.689	0.664	0.648	**0.699**	0.692
H-2-IAk	0.177	0.040	0.240	0.360	0.128	0.055	**0.402**	0.356
H-2-IAs	0.569	0.460	0.543	0.577	0.547	0.484	**0.580**	0.563
H-2-IAu	0.553	0.500	0.576	0.543	0.518	0.509	0.536	**0.590**
H-2-IEd	0.520	0.439	0.508	0.459	**0.543**	0.442	0.476	0.456
H-2-IEk	0.749	0.727	0.717	0.711	0.712	0.712	0.725	**0.750**
DPA1*0103-DPB1*0201	0.748	0.701	0.752	0.764	0.746	0.703	0.761	**0.762**
DPA1*0103-DPB1*0301	0.770	0.741	0.786	0.795	0.769	0.744	0.797	**0.799**
DPA1*0103-DPB1*0401	0.885	0.876	0.892	**0.893**	0.884	0.873	0.892	0.890
DPA1*0103-DPB1*0402	0.473	0.420	0.511	**0.548**	0.486	0.449	0.521	0.509
DPA1*0103-DPB1*0601	0.959	0.954	0.956	0.958	**0.962**	0.953	0.958	0.958
DPA1*0201-DPB1*0101	0.844	0.833	**0.852**	0.851	0.843	0.834	0.851	0.850
DPA1*0201-DPB1*0501	0.811	0.794	0.809	**0.823**	0.809	0.794	0.820	0.821
DPA1*0201-DPB1*1401	0.858	0.835	0.860	0.863	0.855	0.835	0.866	**0.864**
DPA1*0301-DPB1*0402	0.837	0.826	0.844	**0.848**	0.834	0.827	0.847	0.845
DQA1*0101-DQB1*0501	0.779	0.727	**0.805**	0.798	0.772	0.728	0.797	0.797
DQA1*0102-DQB1*0501	**0.658**	0.547	0.618	0.614	0.647	0.518	0.616	0.617
DQA1*0102-DQB1*0502	0.656	0.552	**0.691**	0.690	0.650	0.553	0.679	0.672
DQA1*0102-DQB1*0602	0.795	0.769	0.822	0.825	0.791	0.762	0.825	**0.827**
DQA1*0103-DQB1*0603	**0.605**	0.514	0.601	0.573	0.599	0.537	0.597	0.597
DQA1*0104-DQB1*0503	0.598	0.486	0.667	**0.675**	0.593	0.481	0.667	0.659
DQA1*0201-DQB1*0202	0.625	0.568	0.656	0.661	0.621	0.572	**0.662**	0.656
DQA1*0201-DQB1*0301	0.676	0.634	0.684	0.695	0.675	0.637	0.696	**0.697**
DQA1*0201-DQB1*0303	0.723	0.696	0.745	**0.752**	0.711	0.699	0.747	0.748
DQA1*0201-DQB1*0402	0.569	0.499	0.650	0.653	0.570	0.497	0.655	**0.656**
DQA1*0301-DQB1*0301	0.620	0.547	0.640	0.657	0.643	0.541	0.650	**0.664**
DQA1*0301-DQB1*0302	0.674	0.634	**0.695**	0.684	0.673	0.629	0.692	0.690
DQA1*0303-DQB1*0402	0.538	0.345	**0.590**	0.580	0.548	0.321	0.578	0.579
DQA1*0401-DQB1*0402	**0.748**	0.708	0.731	0.745	0.741	0.713	0.744	0.743
DQA1*0501-DQB1*0201	0.762	0.728	0.776	0.776	0.765	0.734	**0.778**	0.776
DQA1*0501-DQB1*0301	0.797	0.754	0.813	0.813	0.789	0.748	**0.816**	0.815
DQA1*0501-DQB1*0302	0.652	0.613	0.652	0.654	0.648	0.627	**0.658**	0.655
DQA1*0501-DQB1*0303	0.704	0.677	0.702	**0.719**	0.699	0.684	0.707	0.703
DQA1*0501-DQB1*0402	0.659	0.572	0.678	0.687	0.651	0.566	0.681	**0.682**
DQA1*0601-DQB1*0402	0.484	0.332	**0.531**	0.516	0.478	0.325	0.516	0.521
Average	0.679	0.634	0.689	0.693	0.675	0.633	**0.694**	0.692

Table A5 Detail Performance (SRCC) of using different positional encoding conditions on BD2016.

Allele	Calibrated PE				Direct PE			
	Pre (O)	Pre (T)	Post (O)	Post (T)	Pre (O)	Pre (T)	Post (O)	Post (T)
DRB1*0101	0.697	0.682	0.684	**0.713**	0.694	0.675	0.711	0.711
DRB1*0103	0.324	**0.345**	0.317	0.242	0.241	0.321	0.211	0.209
DRB1*0301	0.613	0.582	0.625	**0.643**	0.607	0.576	0.641	0.639
DRB1*0401	0.632	0.606	0.633	**0.646**	0.628	0.599	0.645	0.641
DRB1*0402	0.461	0.561	0.506	**0.568**	0.506	0.519	0.492	0.478
DRB1*0403	**0.708**	0.637	0.647	0.601	0.698	0.645	0.624	0.624
DRB1*0404	0.669	0.646	0.661	0.674	0.665	0.640	**0.679**	0.672
DRB1*0405	0.673	0.646	0.682	0.689	0.671	0.639	**0.691**	0.689
DRB1*0701	0.754	0.735	0.766	**0.774**	0.751	0.733	0.772	0.771
DRB1*0801	0.691	0.646	0.699	0.710	0.687	0.641	**0.711**	0.708
DRB1*0802	0.656	0.638	0.659	**0.664**	0.661	0.639	0.662	**0.664**
DRB1*0901	0.673	0.660	0.682	0.681	0.672	0.659	**0.684**	0.681
DRB1*1001	0.766	0.747	0.767	0.763	0.766	0.737	**0.769**	0.765
DRB1*1101	0.743	0.714	0.745	**0.750**	0.743	0.708	**0.750**	0.746
DRB1*1201	0.754	0.711	0.767	**0.771**	0.753	0.710	0.768	0.766
DRB1*1301	**0.693**	0.646	0.616	0.663	0.685	0.647	0.668	0.658
DRB1*1302	0.759	0.723	**0.763**	0.758	0.756	0.716	0.761	0.758
DRB1*1501	0.687	0.660	0.693	0.700	0.686	0.653	**0.702**	**0.702**
DRB1*1602	0.791	0.774	**0.795**	0.790	0.790	0.771	0.794	0.794
DRB3*0101	0.728	0.716	0.731	**0.742**	0.722	0.709	0.741	0.740
DRB3*0202	0.722	0.698	0.737	**0.740**	0.722	0.693	0.738	0.735
DRB3*0301	0.691	0.633	**0.718**	0.707	0.669	0.628	0.705	0.700
DRB4*0101	0.662	0.631	0.688	**0.704**	0.661	0.630	0.702	0.702
DRB4*0103	0.629	0.632	0.632	0.643	0.626	0.631	**0.652**	0.642
DRB5*0101	0.734	0.721	0.738	**0.747**	0.730	0.714	**0.747**	0.745
H-2-IAb	0.715	0.688	0.726	**0.735**	0.712	0.690	0.728	0.726
H-2-IAd	0.683	0.639	0.676	0.692	0.667	0.645	**0.704**	0.696
H-2-IAk	0.225	0.005	0.268	0.375	0.158	0.033	**0.414**	0.403
H-2-IAs	0.587	0.476	0.580	0.593	0.578	0.486	**0.598**	0.581
H-2-IAu	0.472	0.449	0.588	0.521	0.435	0.423	0.508	**0.594**
H-2-IEd	0.578	0.513	0.572	0.553	**0.600**	0.509	0.550	0.543
H-2-IEk	**0.650**	0.607	0.539	0.615	0.551	0.550	0.586	0.643
DPA1*0103-DPB1*0201	0.712	0.672	0.711	**0.724**	0.711	0.673	0.719	**0.724**
DPA1*0103-DPB1*0301	0.780	0.759	0.799	**0.807**	0.782	0.758	0.806	**0.807**
DPA1*0103-DPB1*0401	0.819	0.806	0.827	**0.828**	0.820	0.805	0.826	0.826
DPA1*0103-DPB1*0402	0.467	0.388	0.528	0.575	0.478	0.410	**0.536**	0.527
DPA1*0103-DPB1*0601	0.864	0.850	0.855	**0.870**	0.866	0.851	0.868	0.866
DPA1*0201-DPB1*0101	0.788	0.770	**0.799**	0.796	0.789	0.770	0.797	0.797
DPA1*0201-DPB1*0501	0.774	0.761	0.776	**0.792**	0.774	0.759	0.787	0.789
DPA1*0201-DPB1*1401	0.834	0.811	0.838	0.843	0.833	0.813	**0.846**	0.843
DPA1*0301-DPB1*0402	0.804	0.789	0.813	**0.814**	0.802	0.789	0.813	0.812
DQA1*0101-DQB1*0501	0.781	0.734	0.804	0.803	0.777	0.734	0.803	**0.805**
DQA1*0102-DQB1*0501	**0.676**	0.558	0.632	0.629	0.666	0.528	0.634	0.632
DQA1*0102-DQB1*0502	0.645	0.576	**0.685**	**0.685**	0.645	0.574	0.673	0.665
DQA1*0102-DQB1*0602	0.779	0.750	0.810	0.812	0.776	0.744	0.813	**0.815**
DQA1*0103-DQB1*0603	**0.586**	0.494	0.580	0.561	0.578	0.515	0.579	0.583
DQA1*0104-DQB1*0503	0.610	0.530	0.662	**0.665**	0.603	0.528	0.660	0.649
DQA1*0201-DQB1*0202	0.637	0.586	0.662	0.665	0.630	0.589	**0.668**	0.663
DQA1*0201-DQB1*0301	0.685	0.641	0.695	0.703	0.685	0.644	0.706	**0.707**
DQA1*0201-DQB1*0303	0.738	0.714	**0.763**	0.768	0.727	0.716	**0.763**	0.762
DQA1*0201-DQB1*0402	0.573	0.517	0.646	0.642	0.573	0.516	0.646	**0.650**
DQA1*0301-DQB1*0301	0.574	0.513	0.557	0.589	0.600	0.507	0.585	**0.607**
DQA1*0301-DQB1*0302	0.684	0.646	**0.702**	0.691	0.683	0.641	0.698	0.697
DQA1*0303-DQB1*0402	0.519	0.337	**0.563**	0.553	0.531	0.313	0.544	0.554
DQA1*0401-DQB1*0402	**0.748**	0.712	0.729	0.743	0.740	0.716	0.742	0.739
DQA1*0501-DQB1*0201	0.763	0.737	**0.776**	0.775	0.765	0.737	0.775	0.773
DQA1*0501-DQB1*0301	0.794	0.749	0.810	0.811	0.784	0.744	**0.814**	0.812
DQA1*0501-DQB1*0302	0.677	0.633	0.673	0.681	0.669	0.644	**0.682**	0.680
DQA1*0501-DQB1*0303	0.729	0.705	0.730	**0.745**	0.723	0.713	0.731	0.732
DQA1*0501-DQB1*0402	0.687	0.603	0.701	**0.711**	0.679	0.596	0.707	0.709
DQA1*0601-DQB1*0402	0.460	0.325	**0.494**	0.478	0.455	0.318	0.476	0.485
Average	0.672	0.630	0.681	**0.687**	0.666	0.626	0.685	0.686

Appendix C

Table A6 Binding core prediction results of Calibrated PE. Red letters are true cores. Only wrongly predicted cores are shown for each method.

PDB	Allele	Antigen Core	Pre (O)	Pre (T)	Post (O)	Post (T)
1A6A	DRB1*0301	MRMATPLLM		RMATPLLMQ		
3PGD	DRB1*0101	MRMATPLLM			PLLMQALPM	
3PDO	DRB1*0101	MRMATPLLM	KMRMATPLL			
1T5X	DRB1*0101	YSDQATPLL	SDQATPLLL	SDQATPLLL		SDQATPLLL
2SEB	DRB1*0401	MRADAAAGG	YMRADAAAG	YMRADAAAG	YMRADAAAG	
4MCZ	DRB1*0401	YATRSSAVR	VYATRSSAV	VYATRSSAV	VYATRSSAV	VYATRSSAV
4MCY	DRB1*0401	VRLRSSVPG	RLRSSVPGV			
1S9V	DQA1*0505-DQB1*0201	PFPQPELPY	LQPFPQPEL	LQPFPQPEL	QPFPQPELP	LQPFPQPEL
1UVQ	DQA1*0102-DQB1*0602	LPSTKVSWA		TKVSWAAVG		WAAVGGGGS
4GG6	DQA1*0301-DQB1*0302	EGSFQPSQE		SFQPSQENP		
4D8P	DQA1*0301-DQB1*0201	EQPEQPFPQ	QPEQPEQPF	PQPEQPEQP	QPEQPFPQP	PQPEQPEQP
4OZG	DQA1*0505-DQB1*0201	PQPELPYPQ	APQPELPYP	APQPELPYP		APQPELPYP
4P5M	DPA1*0103-DPB1*0201	YDGKDYIAL		QAYDGKDYI		
4P23	H-2-IAb	AQKAKANKA		FEAQKAKAN		
Numbers of Correct / Numbers of Total			43/51	40/51	46/51	45/51

Table A7 Binding core prediction results of Direct PE. Red letters are true cores. Only wrongly predicted cores are shown for each method.

PDB	Allele	Antigen Core	Pre (O)	Pre (T)	Post (O)	Post (T)
2FSE	DRB1*0101	FKGEQGPKG				KGEQGPKGE
1J8H	DRB1*0401	YVKQNTLKL		VKQNTLKLA		VKQNTLKLA
1FYT	DRB1*0101	YVKQNTLKL		VKQNTLKLA		VKQNTLKLA
3L6F	DRB1*0101	YEKLSAEQS		EKLSAEQSP		EKLSAEQSP
1A6A	DRB1*0301	MRMATPLLM	RMATPLLMQ	RMATPLLMQ	RMATPLLMQ	RMATPLLMQ
2IPK	DRB1*0101	WVKQNTLKL		VKQNTLKLA		VKQNTLKLA
1SJH	DRB1*0101	VIPMFSALS		IPMFSALSE		IPMFSALSE
3QXA	DRB1*0101	MRMATPLLM		RMATPLLMQ	RMATPLLMQ	RMATPLLMQ
3PGD	DRB1*0101	MRMATPLLM			PLLMQALPM	RMATPLLMQ
3PDO	DRB1*0101	MRMATPLLM				RMATPLLMQ
1AQD	DRB1*0101	WRFLRGYHQ		FLRGYHQYA	FLRGYHQYA	RFLRGYHQY
1PYW	DRB1*0101	FVKQNAAAL		VKQNAAALX		
3C5J	DRB3*0301	IILNHPGQI		ILNHPGQIS		ILNHPGQIS
4OV5	DRB1*0101	ARFLRGYHL	FLRGYHLYA	FLRGYHLYA		
4IS6	DRB1*0401	LYPEWTEAQ		PEWTEAQRL		YPEWTEAQR
4H25	DRB3*0301	IRCNIPKRI		GPSKVATLV		
1SJE	DRB1*0101	VIPMFSALS		FSALSEGAT		IPMFSALSE
1H15	DRB5*0101	YHFVKKHVH				HFVKKHVHE
1T5X	DRB1*0101	YSDQATPLL	SDQATPLLL	DQATPLLLS	DQATPLLLS	DQATPLLLS
1BX2	DRB1*1501	VHFFKNIVT		FFKNIVTPR		
4MD4	DRB1*0401	YRVRVNSAY	VRVNSAYQD	VRVNSAYQD		
4I5B	DRB1*0101	VVKQNCLKL	VKQNCLKLA	VKQNCLKLA	VKQNCLKLA	VKQNCLKLA
4MCZ	DRB1*0401	YATRSSAVR	VYATRSSAV		VYATRSSAV	VYATRSSAV
4MCY	DRB1*0401	VRLRSSVPG	LRSSVPGVR			
1YMM	DRB1*1501	VHFFKNIVT		FKNIVTPRG		
4MDI	DRB1*0402	VRLRSSVPG	LRSSVPGVR			
1S9V	DQA1*0505-DQB1*0201	PFPQPELPY	LQPFPQPEL	QPFPQPELP	QPFPQPELP	LQPFPQPEL
1UVQ	DQA1*0102-DQB1*0602	LPSTKVSWA		TKVSWAAVG	VSWAAVGGG	VSWAAVGGG
4D8P	DQA1*0301-DQB1*0201	EQPEQPFPQ	QPEQPFPQP	QPEQPFPQP	QPEQPEQPF	PQPEQPEQP
4OZG	DQA1*0505-DQB1*0201	PQPELPYPQ	QPELPYPQP	APQPELPYP	APQPELPYP	APQPELPYP
3LQZ	DPA1*0103-DPB1*0201	FHYLPFLPS		HYLPFLPST		
1MUJ	H-2-IAb	MRMATPLLM		MATPLLMQA		
4P23	H-2-IAb	AQKAKANKA		QKAKANKAV		QKAKANKAV
Numbers of Correct / Numbers of Total			40/51	25/51	40/51	27/51
